# Macronutrient-differential dietary pattern impacts on body weight, hepatic inflammation, and metabolism

**DOI:** 10.3389/fnut.2024.1356038

**Published:** 2024-05-29

**Authors:** Yuan-yuan Li, Supradeep S. Madduri, Erika T. Rezeli, Charlene Santos, Herman Freeman III, Jing Peng, Susan L. McRitchie, Wimal Pathmasiri, Stephen D. Hursting, Susan J. Sumner, Delisha A. Stewart

**Affiliations:** ^1^Metabolomics and Exposome Laboratory, Nutrition Research Institute, Department of Nutrition, University of North Carolina at Chapel Hill, Kannapolis, NC, United States; ^2^Department of Nutrition, University of North Carolina at Chapel Hill, Chapel Hill, NC, United States; ^3^Animal Studies Core Lab, Lineberger Comprehensive Cancer Center, University of North Carolina at Chapel Hill, Chapel Hill, NC, United States

**Keywords:** dietary patterns, mouse model, inflammatory biomarkers, cytokines, metabolic biomarkers, metabolomics, macronutrients

## Abstract

**Introduction:**

Obesity is a multi-factorial disease frequently associated with poor nutritional habits and linked to many detrimental health outcomes. Individuals with obesity are more likely to have increased levels of persistent inflammatory and metabolic dysregulation. The goal of this study was to compare four dietary patterns differentiated by macronutrient content in a postmenopausal model. Dietary patterns were high carbohydrate (HC), high fat (HF), high carbohydrate plus high fat (HCHF), and high protein (HP) with higher fiber.

**Methods:**

Changes in body weight and glucose levels were measured in female, ovariectomized C57BL/6 mice after 15 weeks of feeding. One group of five mice fed the HCHF diet was crossed over to the HP diet on day 84, modeling a 21-day intervention. In a follow-up study comparing the HCHF versus HP dietary patterns, systemic changes in inflammation, using an 80-cytokine array and metabolism, by untargeted liquid chromatography-mass spectrometry (LCMS)-based metabolomics were evaluated.

**Results:**

Only the HF and HCHF diets resulted in obesity, shown by significant differences in body weights compared to the HP diet. Body weight gains during the two-diet follow-up study were consistent with the four-diet study. On Day 105 of the 4-diet study, glucose levels were significantly lower for mice fed the HP diet than for those fed the HC and HF diets. Mice switched from the HCHF to the HP diet lost an average of 3.7 grams by the end of the 21-day intervention, but this corresponded with decreased food consumption. The HCHF pattern resulted in dramatic inflammatory dysregulation, as all 80 cytokines were elevated significantly in the livers of these mice after 15 weeks of HCHF diet exposure. Comparatively, only 32 markers changed significantly on the HP diet (24 up, 8 down). Metabolic perturbations in several endogenous biological pathways were also observed based on macronutrient differences and revealed dysfunction in several nutritionally relevant biosynthetic pathways.

**Conclusion:**

Overall, the HCHF diet promoted detrimental impacts and changes linked to several diseases, including arthritis or breast neoplasms. Identification of dietary pattern-specific impacts in this model provides a means to monitor the effects of disease risk and test interventions to prevent poor health outcomes through nutritional modification.

## Introduction

Over the past 40 years, the percentage of calories consumed from carbohydrates has increased dramatically, while the percentage of calories from fat has decreased in the U.S. (1). This macronutrient trend, translating to an overall increase in total calories, parallels the rise in obesity over the past three decades (1). According to the National Health and Nutrition Examination Survey (NHANES) results through 2018, nearly 80% of all American adults fall into an unhealthy weight classification of having overweight, obesity, or severe obesity (2, 3). Obesity contributes a significant burden on health status and is linked to the development of several chronic diseases and co-morbid conditions (4–6). Although obesity has multi-factorial etiology, including genetic, epigenetic, social, psychological, and environmental determinants, diet is one of the most important (4, 5, 7, 8) because it is modifiable in a way that can improve health outcomes. In recent decades, researchers have pointed out that in addition to overall calories, the types of diet or dietary patterns based on different compositions of macronutrients (carbohydrates- simple versus complex, fats- healthy versus unhealthy, and protein-lean versus processed) play a significant role in obesity status and overall health (1, 9–18). In Westernized societies, the condition of obesity is often associated with overconsumption of energy-dense, ultra-processed, high fat, and/or high *simple/refined* carbohydrate dietary patterns, resulting in increased immune system dysregulation and metabolic dysfunction (19–25). For example, systemic and localized changes in inflammation, especially chronic inflammation (26–29), compounded by metabolic reprogramming (17, 30, 31) in response to certain dietary patterns can promote pro-malignant environments (32–37) and greater risks for developing a myriad of cancer phenotypes (38, 39) or increased cardio-metabolic impairments with cardiovascular damage (40–42). Regulation of energy metabolism is highly influenced by diet (12, 43), and individuals with obesity often present with co-morbid conditions across a spectrum of metabolic syndromes (15, 44, 45). Diet-influenced metabolic dysfunctions, specifically related to insulin resistance and lipid metabolism, have also been shown to play significant roles in Type II Diabetes incidence and complications (46–50). The distinction in carbohydrate type is important to this point, as simple carbohydrates *or sugars* metabolize more rapidly compared to complex carbohydrates *like fiber in whole grains*, contributing to blood sugar spikes and prolonged high blood sugar status (51–53). This is also of importance when considering healthier dietary patterns holistically, as patterns with *nutrient-dense* carbohydrates plus sufficient amounts of healthier protein and fat sources have been shown to be better overall (54). In addition, emerging evidence has linked dietary components and consequential inflammation to obesity-associated fatty liver disease, which can also contribute to reduced drug efficacy, resulting in poorer health outcomes (55–57). Even so, the evidence for the contribution of specific macronutrient levels within the different dietary patterns studied to drive specific detrimental outcomes is vast, but it can be confusing and sometimes controversial. When layering on the presence or absence of obesity and the associated outcomes obesity can contribute to, the picture is even more poorly understood by the general population. We believe that the trend of increased consumption of both unhealthy simple carbohydrates combined with similarly high amounts of poor-quality fat content is a primary contributor to the rise in conditions of overweight and obesity, and not just from high fat or carbohydrate alone. This type of dietary pattern is especially detrimental for women in the postmenopausal state as it relates to elevated risks for chronic diseases.

Our study sought to better define dietary pattern influence by comparing isocaloric differences in matched high macronutrient content for total carbohydrate, fat, and protein, sticking to ratios of 25:30:**45** in three of the diets (HC, HF, and HP). We then formulated a dietary pattern with equally higher percentages of both simple carbohydrate and fat (HCHF, 20:**40**:**40**) content to test whether it would render more detrimental outcomes by comparison. We used a female postmenopausal model with ovariectomized female mice due to its relevance to our other research in obesity-sensitive cancers and the co-morbid condition of Type II Diabetes that impacts women (58). While our results are most reflective of women in the postmenopausal state, we believe some of the metabolic and inflammatory biomarker observations will remain the same in women across their lifespans and in men. Future studies will need to be performed to confirm this. In our two pilot studies, we first compared the effects of four dietary patterns to mimic those that are widely consumed in the U.S. population, including high carbohydrate (HC) macronutrient content (45%), high fat (HF) content (45%), high carbohydrate plus high fat (HCHF) content (40% each), and the other having high protein and higher fiber (HP) content (45%), on food consumption, body weight, and glucose level after 15 weeks of feeding and including a diet intervention from the worse dietary pattern (HCHF) to a better one (HP). We then performed a reproducibility study in a follow-up pilot, focused on the two most divergent dietary patterns, comparing only the HCHF and HP diets, confirming body weight and food composition results, and further determining how these patterns influence inflammatory and metabolic biomarker changes, using a relative detection cytokine array (80 markers) and by untargeted metabolomics, respectively. Finally, we conducted pathway analysis to define the relevant disease outcomes associated with identified metabolic differences. When we initiated our first study, our dietary formulations were novel from those previously published in the current literature, even if they represent the same types of dietary patterns. They were developed after an extensive review of dietary studies using mouse models, where a majority of the studies were performed on male rodents. The HC-only diet served as the control pattern for the study, as most standard chow or defined purified diets are higher in carbohydrate content. The HF-only diet served as an additional comparative control with the same amount of higher fat content (45%) in our initial pilot. We are aware that several dietary studies have reported the impacts of HF diets, for example, using the *diet-induced obesity* (DIO) series of diets by Research Diets, Inc. (59); however, we found it necessary to include the HF pattern in our initial study to evaluate the synergistic contribution of higher simple carbohydrate plus higher fat content. At the time of this study, we found no such publications that also compared these three patterns to one that matched high protein content of 45% (HP) with more fiber. The HP diet represents a healthier pattern overall, and no studies have compared these four patterns in a female mouse model representing the postmenopausal condition.

Our results revealed the highly inflammatory nature of the HCHF diet and identified several nutrient-relevant metabolite differences and disease-specific or endogenous pathway perturbations that were distinguished between the HCHF and HP diets. Our results from each study are both unique and complementary, based on the study design, where the four-diet study demonstrates differences in dietary pattern-associated obesity induction and the ability to change body weight health outcome with a short-term nutritional modification (21 days). The two-diet study reproduced weight changes for the HCHF and HP diets, which were the main focus of our study, and pointed to specific inflammation and metabolism mechanistic changes between these dietary patterns, identifying a potential increased risk for five disease outcomes. We hope this research contributes to a greater understanding of which dietary patterns, based on macronutrient differences, are a primary and modifiable exposure linked to specific diseases such as cancer and Type II Diabetes to better model (60) and inform on using nutrition to precisely improve health for women in future human trials.

## Materials and methods

### Diet formulations

The diets used in this study represent novel formulations customized through consultation with Research Diets, Inc., with the ingredients presented in Table 1. Following a review of the literature for dietary patterns used in similar studies, we ultimately used the recommendations from the *Dietary Guidelines for Americans 2015–2020* and *Dietary Reference Intakes: The Essential Guide to Nutrient Requirements* as the basis for our dietary formulations (54, 61). According to these reports, the recommended range of carbohydrate content is 45–65% for humans. Thus, for the HC dietary pattern, we used 45% as the high content amount, which falls within the range. The HC diet serves as the control pattern for the study. The recommendations for humans regarding protein and fat amounts are 15–35% and 20–35%, respectively. Even so, we wanted to match *high* macronutrient levels and used 45% to better compare having greater contents of each primary macronutrient at the same levels in three of the diets (HC, HF, and HP). We then sought to compare those formulations to a dietary pattern that closely reflects the Westernized or Standard American Diet with increased content of both simple carbohydrates and fat (HCHF). We also increased the fiber content (inulin) in the HP formulation as a known optimization in carbohydrate source or quality, hypothesizing that this would make the HP dietary pattern healthier than the other three evaluated patterns. The HC diet was thus formulated with a macronutrient content of total carbohydrate at 45% (complex fiber, 1.5%), fat at 25%, and protein at 30%. The HF diet was formulated with a macronutrient content of total carbohydrates at 25% (complex fiber, 1.5%), fat at 45%, and protein at 30%. The HCHF diet was formulated with a macronutrient content of total carbohydrates at 40% (complex fiber, 1.5%), fat at 40%, and protein at 20%. The HP diet was formulated with a macronutrient content of total carbohydrates at 30% (complex fiber, 5%), fat at 25%, and protein at 45%. Each diet had an equal total kcal percentage of 4,057, although not isocaloric matches by gram amounts, the formulations were developed to closely recapitulate *real-world* macronutrient amounts of a westernized dietary pattern for comparison with a dietary pattern similar to the South Beach Diet; both of which are commonly consumed in the U.S. As customized formulations, Research Diets, Inc. assigned the following catalog numbers to each purified ingredient dietary pattern: HC (#D18101605), HF (#D18101606), HCHF (#D18101607), and HP (#D18101608). Diets were shipped in small batch quantities to either animal facility after heat-sealing but were not irradiated.

**Table 1:** Description of nutritional contents for the four dietary patterns.

Diet group	High Carb (HC)		High Fat (HF)		High Carb + High Fat (HCHF)		High protein (HP)	
%	g	*kcal*	g	*kcal*	g	*kcal*	g	*kcal*
Protein	31	***30***	35	***30***	22.6	***20***	43.8	***45***
Carbohydrate	49	***45***	32.1	***25***	48	***40***	37.3	***30***
Sucrose	31	*30*	11.6	*10*	33.9	*30*	9.7	*10*
Inulin	4.1	***1.5***	4.7	***1.5***	4.5	***1.5***	13	***5***
Fat	11.4	***25***	23.2	***45***	20	***40***	10.8	***25***
Total		*100*		*100*		*100*		*100*
kcal/g	4.13		4.66		4.51		3.89	
Ingredient	**g**	***kcal***	**g**	***kcal***	**g**	***kcal***	**g**	***kcal***
Casein, 80 Mesh	300	*1,200*	300	*1,200*	200	*800*	450	*1800*
L-Cystine	4.5	*18*	4.5	*18*	3	*12*	6.75	*27*
Corn Starch	86.5	*346*	87.5	*350*	36.1	*144.4*	102.29	*409.2*
Maltodextrin 10	50	*200*	50	*200*	50	*200*	50	*200*
Sucrose	295	*1,180*	91.43	*365.7*	295	*1,180*	91.43	*365.7*
Cellulose, BW200	37.5	*0*	37.5	*0*	37.5	*0*	37.5	*0*
Inulin	40.6	*61*	40.6	*61*	40.6	*61*	135.3	*203*
Soybean Oil	25	*225*	25	*225*	25	*225*	25	*225*
Lard	87.5	*788*	177.5	*1,598*	155	*1,395*	87.5	*788*
Mineral Mix S10026	10	*0*	10	*0*	10	*0*	10	*0*
DiCalcium Phosphate	13	*0*	13	*0*	13	*0*	13	*0*
Calcium Carbonate	5.5	*0*	5.5	*0*	5.5	*0*	5.5	*0*
Potassium Citrate, 1 H_2_O	16.5	*0*	16.5	*0*	16.5	*0*	16.5	*0*
Vitamin Mix V10001	10	*40*	10	*40*	10	*40*	10	*40*
Choline Bitartrate	2	*0*	2	*0*	2	*0*	2	*0*
FD&C Yellow Dye #5	0.01	*0*	0	*0*	0.005	*0*	0	*0*
FD&C Red Dye #40	0	*0*	0.01	*0*	0	*0*	0	*0*
FD&C Blue Dye #1	0	*0*	0	*0*	0.005	*0*	0.01	*0*
Total kcal%	**983.61**	***4,057***	**871.04**	***4,057***	**899.21**	***4,057***	**1042.78**	***4,057***

Gram (g), kilocalorie (kcal), water (H_2_O), Food, Drug & Cosmetic (FD&C), and percent (%). The V10001 is from the Research Diets, Inc. AIN-76A Vitamin Mix and the S10026 is from their RD-96 Mineral Mix without calcium, phosphorous, and potassium. The HP dietary pattern has 30% higher complex carbohydrate or fiber content (inulin = 5 kcal) compared to the other three dietary patterns (inulin = 1.5 kcal).

### *In vivo* pilot studies

**Figure 1:**
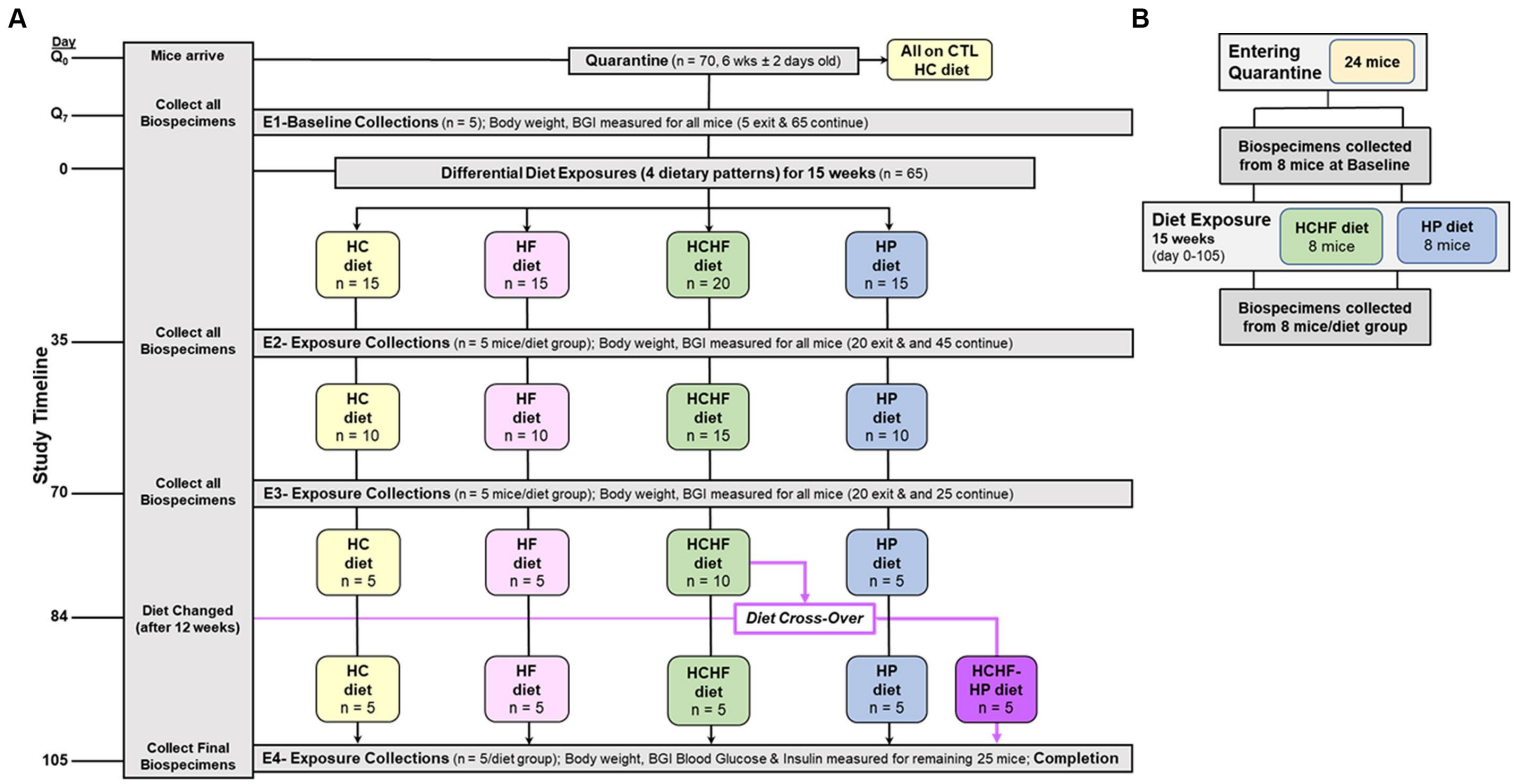
Schematics of 15-week dietary pattern comparative *in vivo* studies. **(A)** Four-diet study was conducted as follows: (+ 1-week of quarantine) used 70 6-week old (± 2 days) ovariectomized female C57BL/6 mice. Multi-organ and other biospecimen collections occurred at endpoints (E1–E4) corresponding to Days Q7 (Baseline), 35, 70, and 105 (Completion). At Day 84, half of the remaining mice fed the HCHF diet were changed over to the HP diet for the remaining 3 weeks (new group designated as HCHF→HP). BG was measured at these 4 study timepoints. Body weight and food consumption were measured for all mice before endpoint (E1–E4) biospecimen collections and one time weekly for any mouse remaining in study. Food consumption on Day 0, for the cage, and at the end of each week, including a record of the # of mice remaining in the cage. Q, quarantine; CTL, control; BG, Blood glucose; HC, high carbohydrate; HF, high fat; HCHF, high carbohydrate + high fat, and HP, high protein with higher fiber. **(B)** Two-diet study again used 6-week-old, ovariectomized female C57BL/6 mice placed into quarantine for 1 week, and then baseline biospecimens were collected from 8 mice. The remaining 16 mice were randomized to either the HCHF diet group or the HP diet and fed for 15 weeks (105 days). Body weight and food consumption measurements were taken as in the 4-diet study. After study completion, 8 mice from each dietary group were sacrificed and biospecimens were collected. Liver samples were used for inflammation and metabolic marker analyses.

The four-diet *in vivo* study was conducted according to local, state, and federal regulations, and all animal experiments were performed under an approved protocol by the Institutional Animal Care and Use Committee at Explora BioLabs (San Diego, CA), a subsidiary of Charles River Laboratories. The study design is outlined in Figure 1A, where 6-week-old female C57BL/6 mice (*n* = 70, purchased from Charles River Labs, ovariectomized before shipping) were fed the HC diet and had access to food and water *ad libitum* upon arrival to acclimate during quarantine (1 week). Mice were ovariectomized to mimic a postmenopausal state characterized by a reduction in circulating estrogen, bone mineral density loss, and termination of the estrous cycle (62, 63). Following quarantine, five mice were sacrificed to collect baseline biospecimens, and the remaining 65 mice were randomized into one of the four diet groups (*n* = 15/group) for the remainder of the study for the 15 weeks of differential diet exposure to the HC, HF, HCHF, or HP diets. Five mice were grouped in a cage for each diet group, corresponding to three cages/groups for the HC, HF, and HP dietary patterns. The HCHF diet group had 5 extra mice assigned to this dietary pattern (n = 20) to carry out a diet cross-over intervention, as described in Figure 1A on Day 84. Thus, the HCHF dietary pattern had four cages at the start of the study. All results are presented with Day 0 set as the start of the differential dietary feeding period, and at the end of three 5-week feeding periods up to 15 weeks, 5 mice per dietary group were sacrificed after endpoint measurement (body weight, food consumption, and blood glucose measured by standard ELISA) to collect biospecimens. Endpoint biospecimen collections included plasma, buffy coat, urine, feces, cecum, brain, brown adipose tissue, white adipose tissue, abdominal skin flap, kidneys, liver, lungs, pancreas, spleen, uterus, right quadriceps, and mammary gland. All collected biospecimens were immediately stored at −80°C following harvest. The two-diet *in vivo* study was conducted according to local, state, and federal regulations, and all animal experiments were performed under an approved University of North Carolina at Chapel Hill Institutional Animal Care and Use Committee protocol. According to the study design outlined in Figure 1B, 6-week-old female C57BL/6 mice (*n* = 24, purchased from Charles River Labs, ovariectomized before shipping) were fed the HP diet and had access to food and water *ad libitum* upon arrival to acclimate during quarantine (1 week). Following quarantine, eight mice were sacrificed to collect baseline biospecimens, and the remaining 16 mice were randomized into one of the two diet groups (*n* = 8/group) for the remainder of the study for the 15 weeks of differential diet exposure to the HCHF or HP diet. Four mice were grouped in a cage for each diet group, corresponding to two cages/group. All results are presented with Day 0 set as the start of the differential dietary feeding period, and at the end, all 8 mice per diet group were sacrificed to collect biospecimens to evaluate systemic changes. Collected biospecimens for this study included plasma, serum, urine, feces, brain, liver, and mammary gland tissues from the 4th mammary fat pad. All collected biospecimens were immediately stored at −80°C following harvest. As the primary metabolic organ, liver tissue was selected to measure inflammatory and metabolic diet-dependent changes.

For both pilot studies, no special deviations from normal were made for facility conditions, which were under-regulated environmental conditions with the temperature at 25°C and humidity at 50%, with a 12:12-h light–dark cycle. Food consumption was measured weekly per cage, and the amount of food consumed was divided by the number of mice per cage and per day for each measurement, then averaged for all the cages in the diet group (HCHF or HP) for that measurement. Body weight was monitored weekly. For both studies, the method of euthanasia used was cervical dislocation, in consideration of evaluating metabolic changes and knowing that anesthesia would alter the metabolome. In addition, mice scheduled to be exited from either study at different timepoints were fasted for 8 h before euthanasia and biospecimen collections. All applicable international, national, and/or institutional guidelines for the care and use of animals were followed. All procedures performed in studies involving animals were in accordance with the ethical standards and were approved by the internal Explora BioLabs, Inc. Institutional Animal Care and Use Committee (IACUC) and the University of North Carolina at Chapel Hill IACUC under protocol # 18–321.0.

### Inflammatory profiling

Inflammation response was measured by cytokine profiling analysis, performed using G5 series arrays to profile 80 markers (RayBiotech Life, Peachtree Corners, GA). Liver samples (per mouse) were placed in tubes containing homogenization beads and homogenized with 1X lysis buffer (RayBiotech) according to weight. Samples were centrifuged, and supernatants were transferred to clean tubes. Total protein was quantitated using the Pierce BCA assay (Thermo Fisher Scientific, Waltham, MA), and then array slides were prepared for overnight sample hybridization (2.0 μg/sample). After washing and secondary antibody hybridizations, array slides were scanned using 2-color fluorescent detection and the raw data was extracted to yield relative fluorescence units (RFUs) for each of the 80 cytokines per sample. Positive control spots on each array were evaluated to ensure they fell within a quality control range of ≤15% variation to positive control RFUs on a reference-designated array, according to the manufacturer’s analysis procedures. Once determined to pass the quality control criterion across all arrays, the data were preprocessed using the RayBiotech Analysis Tool for background subtraction and normalization to the positive control values on the reference array. Relative fold-changes were calculated in Excel from RFUs of HCHF-fed mice (15 weeks) normalized to baseline mice (fed HP diet for 1 week during quarantine), HP-fed mice (15 weeks) normalized to baseline mice (fed HP diet for 1 week during quarantine), and HCHF-fed mice normalized to RFUs of HP-fed mice (both after 15 weeks) for diet-only changes. According to the manufacturer’s thresholds, cytokines with a fold difference of ≥1.5-fold were accepted as significantly upregulated and denoted by bold lettering in Table 2, while cytokines with a fold difference of ≤0.65 were accepted as significantly *downregulated* and denoted by *italicized lettering.*

**Table 2:** Diet-dependent cytokine fold-changes.

CYTOKINES	HCHF *vs* baseline	HP *vs* baseline	HCHF *vs* HP
ENA-78	**438.42**	1.00	**437.93**
GCSF	**10.56**	**1.76**	**5.99**
GM-CSF	**1197.23**	**36.36**	**32.93**
GRO	**96.21**	**2.57**	**37.44**
GRO-a	**17.67**	1.44	**12.31**
I-309	1.39	*0.11*	**12.71**
IL-1a	**3.62**	1.37	**2.65**
IL-1b	**169.54**	1.00	**169.35**
IL-2	**2.72**	*0.01*	**445.88**
IL-3	**13.63**	**2.18**	**6.24**
IL-4	**31.24**	1.00	**31.20**
IL-5	**588.68**	**41.46**	**14.20**
IL-6	**6.09**	**1.59**	**3.83**
IL-7	**21.95**	**2.79**	**7.87**
IL-8	**1609.57**	**74.01**	**21.75**
IL-10	**685.53**	1.00	**684.76**
IL-12 p70	**316.65**	1.00	**316.29**
IL-13	**30.99**	**4.68**	**6.62**
IL-15	**1541.14**	**66.94**	**23.02**
IFN-g	**6.13**	**1.62**	**3.77**
MCP-1	**2.09**	1.04	**2.02**
MCP-2	**384.47**	1.00	**384.04**
MCP-3	**13.18**	*0.11*	**117.06**
MCSF	**2.45**	1.48	**1.66**
MDC	**91.64**	1.00	**91.54**
MIG	**3.18**	1.11	**2.88**
MIP-1b	**306.73**	1.00	**306.38**
MIP-1d	**58.19**	*0.01*	**5553.28**
RANTES	**53.82**	1.00	**53.76**
SCF	**136.11**	1.00	**135.96**
SDF-1	**16.41**	*0.40*	**41.24**
TARC	**19.12**	1.00	**19.10**
TGF-b1	**6.00**	1.29	**4.64**
TNF-a	**2.64**	1.28	**2.07**
TNF-b	**4.31**	0.90	**4.81**
EGF	**2.31**	1.04	**2.22**
IGF-I	**342.86**	1.00	**342.48**
Angiogenin	**3357.74**	1.00	**3353.97**
Oncostatin M	**2.70**	1.05	**2.57**
Thrombopoietin	**74.92**	1.00	**74.84**
VEGF	**51.80**	**6.01**	**8.62**
PDGF-BB	**41.19**	1.00	**41.14**
Leptin	**298.09**	1.00	**297.75**
BDNF	**4.63**	**1.57**	**2.94**
BLC	**1706.32**	**10.87**	**156.92**
Ck b 8–1	**80.68**	*0.06*	**1353.68**
Eotaxin	**608.08**	1.00	**607.40**
Eotaxin-2	**39.82**	1.00	**39.78**
Eotaxin-3	**309.53**	1.00	**309.18**
FGF-4	**333.09**	1.00	**332.71**
FGF-6	**2.93**	1.28	**2.29**
FGF-7	**258.58**	1.00	**258.29**
FGF-9	**396.24**	0.94	**419.92**
Flt-3 Ligand	**2661.21**	**65.60**	**40.57**
Fractalkine	**8.39**	*0.11*	**73.39**
GCP-2	**818.98**	1.00	**818.06**
GDNF	**34.84**	**2.51**	**13.88**
HGF	**614.93**	**4.66**	**131.87**
IGFBP-1	**2.81**	0.74	**3.79**
IGFBP-2	**1.83**	0.77	**2.37**
IGFBP-3	**7.49**	**2.01**	**3.73**
IGFBP-4	**64.33**	1.00	**64.26**
IL-16	**55.94**	1.00	**55.88**
IP-10	**765.67**	1.00	**764.81**
LIF	**509.74**	1.00	**509.17**
LIGHT	**1008.32**	**227.32**	**4.44**
MCP-4	**171.98**	**5.75**	**29.93**
MIF	**384.89**	1.00	**384.45**
MIP-3a	**207.17**	*0.13*	**1560.56**
NAP-2	**1416.94**	**3.76**	**377.06**
NT-3	**314.60**	**3.85**	**81.72**
NT-4	**7.95**	1.35	**5.88**
Osteopontin	**2.98**	1.49	**2.00**
Osteoprotegerin	**39.63**	1.00	**39.59**
PARC	**206.79**	0.78	**266.77**
PIGF	**416.46**	1.00	**415.99**
TGF- b2	**3.49**	**1.65**	**2.11**
TGF- b3	**68.44**	1.00	**68.36**
TIMP-1	**1617.49**	**51.25**	**31.56**
TIMP-2	**188.68**	1.00	**188.47**

Differences were calculated to evaluate changes in the high carbohydrate + high fat (HCHF) group normalized by high protein with higher fiber (HP) diet group after 15 weeks of differential diet exposure (third column). The initial columns are for individual (age-dependent) inflammatory responses where fold-changes were calculated for differential dietary exposures to HCHF or HP diets. Relative fluorescence units (RFUs) for each cytokine across all samples analyzed at the end of 15 weeks were normalized to liver samples collected at baseline (following 1 week in quarantine). The HCHF diet generated a more pro-inflammatory profile compared to the HP diet. Cytokines are significantly upregulated if ≥ 1.5-fold (bold print) or significantly downregulated if ≤ 0.65-fold (italicized print). Non-significant fold changes appear as grayed-out text. Cytokines abbreviations are as follows: ENA-78 (C-X-C motif chemokine ligand 5, CXCL5), GCSF (colony stimulating factor 3), GM-CSF (granulocyte-macrophage colony-stimulating factor 2), GRO (Growth-regulated alpha/beta/gamma protein), GRO-a (C-X-C motif chemokine ligand 1, CXCL1), I-309 (C-C motif chemokine 1, CCL1), IL-1a (Interleukin-1 alpha), IL-1b (Interleukin-1 beta), IL-2 (Interleukin-2), IL-3 (Interleukin-3), IL-4 (Interleukin-4), IL-5 (Interleukin-5), IL-6 (Interleukin-6), IL-7 (Interleukin-7), IL-8 (Interleukin-8, C-X-C motif chemokine 8), IL-10 (Interleukin-10), IL-12 p70 (Interleukin-12 subunit alpha), IL-13 (Interleukin-13), IL-15 (Interleukin-15), IL-16 (Pro-interleukin-16), IFN-g (Interferon gamma), MCP-1 (Monocyte chemoattractant protein 1, C-C motif chemokine 2), MCP-2 (Monocyte chemoattractant protein 2, C-C motif chemokine 8), MCP-3 (Monocyte chemoattractant protein 3, C-C motif chemokine 7), MCSF (Macrophage colony-stimulating factor 1), MDC (Macrophage-derived chemokine, C-C motif chemokine 22), MIG (Monokine induced by interferon-gamma, C-X-C motif chemokine 9), MIP-1b (Macrophage inflammatory protein 1-beta, C-C motif chemokine 4), MIP-1d (Macrophage inflammatory protein 5, C-C motif chemokine 15), RANTES (Regulated upon Activation, Normal T Cell Expressed and Presumably Secreted; C-C motif chemokine 5), SCF (Stem cell factor, Kit ligand), SDF-1 (Stromal cell-derived factor 1, C-X-C motif chemokine 12), TARC (Thymus and activation-regulated chemokine, C-C motif chemokine 17), TGF-b1 (Transforming growth factor beta-1 proprotein), TNF-a (Tumor necrosis factor-alpha), TNF-b (Tumor necrosis factor-beta, Lymphotoxin-alpha), EGF (Pro-epidermal growth factor), IGF-I (Insulin-like growth factor I), ANG (Angiogenin), OSM (Oncostatin M), TPO (Thrombopoietin), VEGF (Vascular endothelial growth factor A, long form), PDGF-BB (Platelet-derived growth factor subunit B), LEP (Leptin), BDNF (Brain-derived neurotrophic factor), BLC (B cell lymphocyte chemoattractant, C-X-C motif chemokine 13), Ck b 8–1 (CK-beta 8, Macrophage inflammatory protein 3, C-C motif chemokine 23), Eotaxin (Eosinophil chemotactic protein, C-C motif chemokine 11), Eotaxin-2 (Eosinophil chemotactic protein 2, C-C motif chemokine 24), Eotaxin-3 (Macrophage inflammatory protein 4-alpha, C-C motif chemokine 26), FGF-4 (Fibroblast growth factor 4, Heparin secretory-transforming protein 1), FGF-6 (Fibroblast growth factor 6, Heparin-binding growth factor 6), FGF-7 (Fibroblast growth factor 7, Heparin-binding growth factor 7, Keratinocyte growth factor), FGF-9 (Fibroblast growth factor 9, Glia-activating factor, Heparin-binding growth factor 9), Flt-3 Ligand (Fms-related tyrosine kinase 3 ligand), Fractalkine (C-X3-C motif chemokine 1), GCP-2 (C-X-C motif chemokine 6), GDNF (Glial cell line-derived neurotrophic factor), HGF (Hepatocyte growth factor), IGFBP-1 (Insulin-like growth factor-binding protein 1), IGFBP-2 (Insulin-like growth factor-binding protein 2), IGFBP-3 (Insulin-like growth factor-binding protein 3), IGFBP-4 (Insulin-like growth factor-binding protein 4), IP-10 (10 kDa interferon gamma-induced protein, C-X-C motif chemokine 10), LIF (Leukemia inhibitory factor), LIGHT (Tumor necrosis factor ligand superfamily member 14), MCP-4 (Monocyte chemoattractant protein 4, C-C motif chemokine 13), MIF (Macrophage migration inhibitory factor), MIP-3a (Macrophage inflammatory protein 3 alpha, C-C motif chemokine 20), NAP-2 (Neutrophil-activating peptide-2, Platelet basic protein, C-X-C motif chemokine 7), NT-3 (Neurotrophin-3, Nerve growth factor 2), NT-4 (Neurotrophin-4), OPN (Osteopontin, Secreted phosphoprotein 1), OPG (Osteoprotegerin, Tumor necrosis factor receptor superfamily member 11B), PARC (Pulmonary and activation-regulated chemokine, Macrophage inflammatory protein 4, C-C motif chemokine 18), PIGF (Placental growth factor), TGF- b2 (Transforming growth factor beta-2 proprotein), TGF- b3 (Transforming growth factor beta-3 proprotein), TIMP-1 (Tissue inhibitor of metalloproteinases 1, Metalloproteinase inhibitor 1), and TIMP-2 (Tissue inhibitor of metalloproteinases 2, Metalloproteinase inhibitor 2).

### Untargeted UPLC high-resolution mass spectrometry metabolomics

#### Sample preparation and data acquisition

Metabolomics analysis was performed after the preparation of liver tissue homogenates (n = 24 mice). In brief, each biospecimen was mixed with ice-cold methanol (10 μL for every mg of tissue) and homogenized with a Bead Ruptor Elite Bead Mill Homogenizer (OMNI International) at 5.0 m/s for 30 s in two cycles. The supernatants were collected after centrifugation at 16,000 rcf for 20 min at 4°C. A quality control pool (QC pool) sample was prepared by pooling 30 μL supernatant aliquots from individual samples (*n* = 24) with a total pre-processing weight ≥ 60 mg. The supernatants (200 μL) of each study sample and QC pools were transferred to new tubes and dried under Speed-vac. Each dried sample was reconstituted in a 200-μL water–methanol (95:5) solution containing 500 ng/mL L-tryphotophan-d5. A 5-μL aliquot of each reconstituted supernatant was used for metabolomics analysis via a Vanquish UHPLC system coupled to a Q Exactive™ HF-X Hybrid Quadrupole-Orbitrap Mass Spectrometer (Thermo Fisher Scientific, San Jose, CA) with conditions described previously (64). Blanks (no tissue samples) were also prepared under identical conditions as the study samples. Samples were run in two batches with a randomized order, with the QC pools and blanks interspersed.

#### Data preprocessing and quality control

Progenesis QI (version 2.2, Waters Corporation) was used for alignment, peak picking, and deconvolution. Background peaks not important to the biological samples were filtered out if the fold changes of these individual peaks between QC pools and blanks were < 3.0 by mean. Peaks were normalized in Progenesis QI using the “normalize to all” feature. Peaks that significantly varied (*q* < 0.05) between the two batches of QC pools were further excluded. After data processing and quality control procedures, 5,538 peaks were retained for further analyses.

### Statistical analyses

#### Food consumption, body weight, and blood glucose

**Figure 2:**
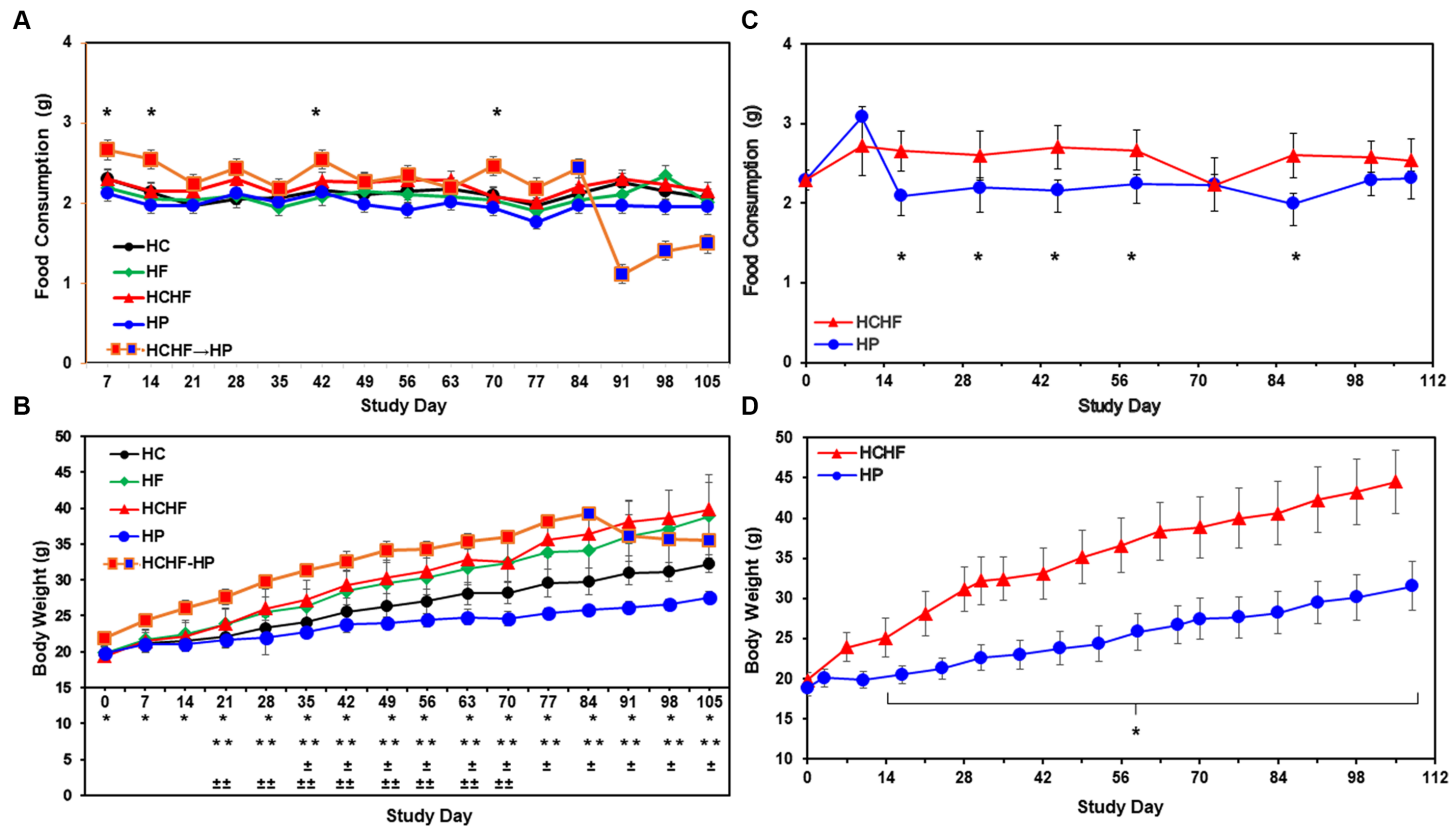
Results from the four-dietary pattern and two-dietary pattern studies after 15 weeks. **(A)** Average daily consumption of food is presented per mouse per day. Following quarantine, mice were fed either the HC, HF, HCHF, or HP diets for 15 weeks (*n* = 5 mice/diet group). One group of mice fed the HCHF diet for 12 weeks was crossed over to the HP diet for the remaining 3 weeks (orange box changes to blue box on Day 84). Error bars are based on the standard error of the average of food consumed per time-point/diet. No significant differences between the HC diet and any other diet in pair-wise *t*-tests. Significance was found on 4 days between the HCHF→HP and HP, *p* < 0.05 (*) by *t*-test, but not rigorous enough to detect significance after Day 84. **(B)** Average body weight change results from four-dietary pattern 15-week study. Differences in weight gain were significant between mice fed the HC diet vs. all other diets and for mice fed the HP diet with all other diets. Error bars are based on standard deviation. Significant differences determined by *t*-test at *p* < 0.001 as follows: HCHF→HP vs. HP (*), HC vs. HCHF and HCHF vs. HP (**), HC vs. HP (^**±**^) and HC vs. HF (^**±±**^). **(C)** Average daily consumption of food is presented per mouse per day. Following quarantine, mice were fed either the HP or HCHF diet for 15 weeks (*n* = 8 mice/diet group). Error bars are based on the standard deviation of average food consumed per mouse/per day at each time point for both diets. Significance (HCHF vs. HP), at least *p* < 0.0001 (*), by *t*-test. **(D)** Average body weight changes in the two-dietary pattern follow-up study. Differences in weight gain were significant between the two dietary groups after the second week of the study. Body weight changes continued to increase throughout the study, with no plateau observed for either diet group. Error bars are based on the standard error of the average of food consumed per time-point/diet. Significance (HCHF vs. HP), *p* < 0.0001 (*), by *t*-test. HC, high carbohydrate; HF, high fat; HCHF, high carbohydrate + high fat; HCHF→HP, high carbohydrate + high fat crossed over to high protein with higher fiber; HP, high protein with higher fiber.

**Figure 3:**
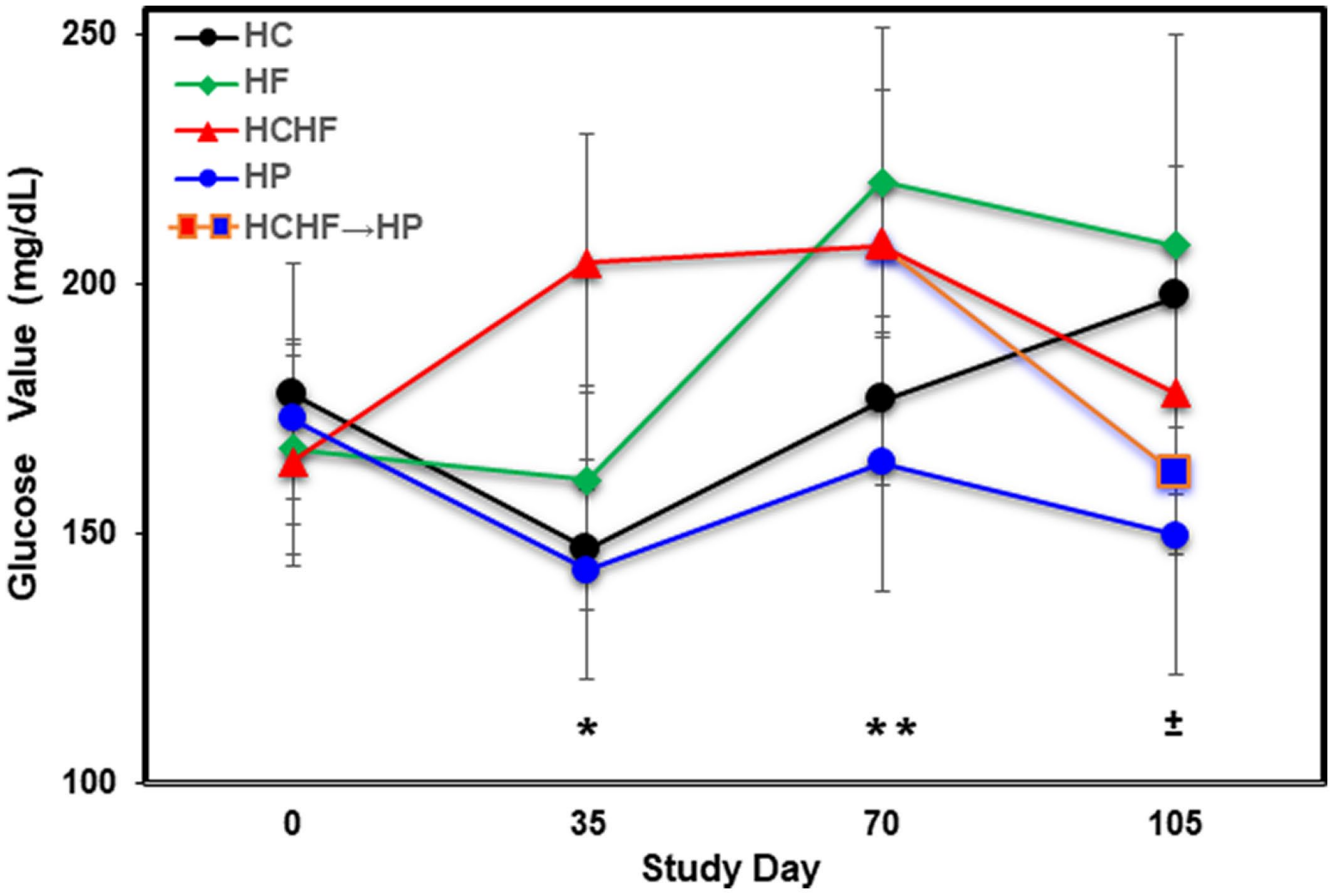
Averaged blood glucose levels (mg/dL) in five mice per dietary pattern measured every 5 weeks over 15 weeks on Days 0, 35, 70, and 105. Mice on the HCHF diet were switched to the HP diet on Day 84, denoted by HCHF→HP. Error bars based on standard deviation. Differences in glucose levels were determined significant by *t*-test on Day 35 between mice fed the HC vs. HCHF diets and the HP vs. HCHF diets (*, *p* < 0.001), on Day 70 between mice fed the HC vs. HF diets and the HCHF vs. HP diets (^**±**^, *p* < 0.05), and on Day 105 between mice fed the HC vs. HP and HCHF→HP diets, and between the HF vs. HP diets (^**^, *p* < 0.05). Error bars are based on standard deviation. HC, high carbohydrate; HF, high fat; HCHF, high carbohydrate + high fat; HCHF→HP, high carbohydrate + high fat crossed over to high protein with higher fiber; HP, high protein with higher fiber.

Descriptive statistics and hypothesis testing using *t*-tests for two independent groups assuming unequal variance were conducted in Microsoft Excel 2016 for samples for the HC versus the other three dietary patterns and the HCHF versus the HP diet. *p*-values <0.05 are considered statistically significant but were not adjusted for multiple testing. Before statistical analysis, food consumption was measured by cage weekly and divided by the number of mice in the cage and the number of days to calculate the amount of food consumed per mouse/per day. This amount was then averaged for each dietary pattern. Similarly, the body weight of each mouse was recorded weekly for all mice. Weights for all mice in the same dietary pattern group were averaged to more easily see the trend across the diets. If significantly different, the *p*-value is denoted in the graphs for Figures 2, 3 for both 15-week pilot studies.

#### Metabolomics

Statistical analyses were conducted using SAS 9.4 (SAS Institute Inc., Cary, NC). Hypothesis tests were conducted using a two-sided *t*-test with the Satterthwaite correction for unequal variances or the Wilcoxon Rank Order Sum test. *p*-values <0.05 were considered statistically significant and were not adjusted for multiple testing. Multivariate data analysis using the normalized untargeted metabolomics data was conducted using SIMCA 16.0 (Umetrics, Umeå, Sweden). Analyses included both unsupervised principal component analysis (PCA) and supervised orthogonal partial least squares discriminate analysis (OPLS-DA). Variable influence on projection (VIP) plots was examined, and the VIP statistic was used as one of the criteria for differentiating the phenotypic groups in each of the OPLS-DA analyses. All models used 7-fold cross-validation to assess the predictive variation of the model (*Q*^2^). Signals with VIP ≥1.0 with a jack-knife confidence interval that did not include 0 and *p* < 0.05 and magnitude of fold-change ≥2-fold were deemed important to the differentiation of the study phenotype (HCHF diet vs. HP diet). Fold-change is reported for the HCHF diet relative to the HP diet.

#### Signal identification and annotation

Peaks associated with study phenotypes as important were identified and annotated by matching signals to the *in-house* experimental standard library and public database (HMDB, NIST, and MELINE) based on available data for retention time (RT), exact mass (MS), MS/MS fragmentation pattern, and isotopic ion pattern.

### Pathway analyses

#### Pathway analysis for inflammatory profiles

Cytokines that were significantly different between the HFHC diet and HP diet after 15 weeks of feeding were correlated to endogenous and xenobiotic-relevant biological pathways using GeneGo, MetaCore mapping software (Clarivate Analytics, PA). Significant enrichment in pathways was based on a *p*-value <0.05.

#### Pathway enrichment analysis for metabolomics

Pathway enrichment was conducted using Gene Set Enrichment Analysis (GSEA) and Mummichog algorithms (“Peaks to Pathways” module) in MetaboAnalyst 4.0 (65, 66). All features (m/z) remaining after data filtering (>5,000 signals) were entered together with the *p*-value that was calculated for the comparison (HCHF diet vs. HP diet as reference) and controls. A *p*-value cutoff of 0.05 and mass accuracy of 3 ppm were used to select significant features to match against all possible metabolites. All possible metabolites that were matched by m/z were searched in the mouse reference metabolic network (mmu*_k_egg*), and the null distribution of module activities was estimated by using 100 permutations of random lists drawn from the experimental reference feature list. The candidate pathways were based on the similarity of m/z.

#### Biochemical pathway interpretation

Endogenous biochemical pathway interpretation was conducted by assessing the connection between analytes noted to significantly increase or decrease (VIP ≥ 1 or *p* < 0.05 or magnitude of fold change ≥2) between the two diets. The detailed interpretations include an assessment of perturbations for consumption of the HCHF diet versus the HP diet for 15 weeks.

## Results

### Impact of different diets on food consumption, body weight, and glucose level

Compositions of the HC, HF, HCHF, and HP diets are described in Table 1. The schema for both *in vivo* studies is outlined in Figure 1A for the four-diet pilot study and Figure 1B for the two-diet pilot study. During both 15-week-long (105 days) studies, significant differences in food consumption only occurred on 4-to-5 days across the dietary pattern exposures (Figures 2A,C). Overall, mice fed the HCHF diet consumed more food in both studies than mice fed all other diets. In particular, the HCHF group consumed a little more food than the HP group (approximately 2.5 g/mouse/day vs. 2.0 g/mouse/day). In the four-diet study, we observed a significant decrease in consumption for mice fed the HCHF diet initially after being switched to the HP diet, most dramatically seen during the first 7 days after the diet cross-over on Day 84 (Figures 1A, 2A), from 2.4 g (HCHF) to 1.1 g (HP) on average. These mice did begin to consume more food in the final 2 weeks, and by the end of the study, they were consuming ~1.5 g of the HP diet on average. Although this amount was still less compared to the consumption of mice on the other three diets during this period, the daily amount of food eaten by mice switched to the HP diet was more comparable to what mice that were consistently assigned to the HP pattern consumed (1.5 g/day vs. 2.0 g/day, respectively).

The reduced consumption of food by mice participating in the 21-day intervention starting on Study Day 84 corresponded to body weight loss after the switch to the HP diet by Study Day 91 (Figure 2B), which seemed to plateau by Day 105, even though the mice were steadily increasing their consumption. All mice in both studies showed normal body weight increases with age, while only mice consuming the HF and HCHF diets became obese in the four-diet study (Figure 2B) or only the HCHF diet in the two-diet study (Figure 2D). Similarly, in the two-diet follow-up study to compare what we considered the *worse* versus the *best* diet, by day 14, the body weights of HCHF-fed mice were significantly higher (*p* < 0.0001) than those of HP-fed mice, and this difference steadily increased with both age and dietary exposure (Figure 2D). At the end of feeding for 15 weeks in the initial study, mice were 21 weeks old and had average weights of 39.8 g (HCHF) > 38.9 g (HF) > 35.5 g (HCHF→HP) > 32.3 g (HC, *control*) > 27.6 g (HP) on the different diets (Figure 2B). A similar pattern of body weight gain was replicated in the follow-up study where HCHF-fed on average weighted 44.6 g compared to 31.5 g for mice fed the HP diet (Figure 2D). According to a previous publication on average body weight and food intake for different mice strains consuming a standard chow diet (mimicked by our HC diet), female C57BL/6 J mice would weigh approximately 14 g at 6 weeks of age, approximately 21 g by 12 weeks of age (~6–7 g increase) and would consume approximately 2.7 g of food daily (67). In the ovariectomized C57BL/6 female mouse model we used in our four-diet study, mice had initial weights of 18–22 g at 6 weeks of age (20 g for HC group), recapitulating increased fat deposition in the postmenopausal state (58), and on the HC diet (similar to standard chow), they gained an expected ~6 g of weight for their age-range after 6 weeks (Day 42), weighing an average of 25–26 g. Thus, only mice fed the HF and HCHF diets experienced dietary pattern-associated weight gains, becoming obese, and the only consumption-associated weight loss was demonstrated during the intervention of feeding mice the HCHF diet for 12 weeks and then converting them to the HP diet for the last 3 weeks, in the four-diet study. On average, by Day 84 (12 weeks of feeding), mice fed the HCHF diet were 12.6 g heavier than mice fed the HP diet across both studies, corresponding to a greater body weight gain ratio of approximately 1.4-fold for HCHF-exposed mice. Similarly, mice fed the HF diet were approximately 11 g heavier on average, compared to the HP diet-fed mice by Day 84 in the four-diet study.

In addition to food consumption and body weight changes, we measured quantitative levels of glucose by ELISA during the four-diet pilot study to determine differential dietary impacts at Days 35, 70, and 105 (at termination). Figure 3 shows that mice fed the HF diet experienced the highest blood glucose levels by Day 105, followed by mice fed the HC > HCHF > HCHF→HP > HP diets. After the first 5 weeks (Day 35), the HCHF mice had the highest blood glucose measurements, as we expected, which was significantly higher compared to the HC or HP diets (*p* < 0.001). By Day 70, mice fed the HF diet exceeded them and maintained higher levels to the end of the study. Mice fed the HC diet also demonstrated increasing levels of glucose by Day 70, which continued to rise despite their normal gains in body weight and non-significant difference in food consumption but were still significantly lower than in mice fed the HF diet (*p* < 0.05). There was also a significant difference between mice fed the HCHF versus the HP diet (p < 0.05). By Day 105, glucose levels for the HF- and HC-fed mice were significantly higher compared to the HP-fed mice (*p* < 0.03) as expected, and these mice had the lowest levels of blood glucose across the 15 weeks. In the two-diet follow-up study, we further investigated system-level changes in inflammation and metabolism using liver tissue, which represents the major metabolic organ, analyzed by cytokine array and untargeted LCMS metabolomics.

### Differential dietary pattern impacts inflammatory cytokine levels in mice

In the two-diet study, we observed several differences in relative detection levels between the HCHF and HP diets for 80 profiled inflammation markers. After a total of 15 weeks of differential diet exposure, nearly all cytokines measured in liver samples of the HCHF-fed mice were significantly higher (Table 2). In comparison, only 24/80 cytokines were upregulated, and 8/80 were downregulated in the liver tissues of HP-fed mice. According to the manufacturer’s thresholds, a significant change in upregulation or downregulation is ±1.5-fold difference (RayBiotech, Inc. ^®^). The range of change in mice fed the HCHF diet went from 2-fold to >3,357-fold across the measured inflammation markers (both pro- and anti-inflammatory) relative to their levels at baseline versus differences of only up to 227-fold in HP diet-fed mice. These changes reflect both age- and dietary pattern-dependent changes (Table 2). Only levels for the pro-inflammatory cytokine I-309, secreted by activated T-lymphocytes and serving as a monocyte chemoattractant (68), were not significantly different after HCHF diet exposure, compared to baseline (Day 0). In contrast, this cytokine was downregulated in mice fed the HP diet. We also compared 15-week fold-changes in the inflammatory levels for mice on the HCHF diet relative to the mice on the HP diet, finding that all 80 inflammation markers were significantly upregulated in HCHF-fed mice over HP-fed mice from 1.66-fold greater [in macrophage colony-stimulating factor (MCSF)/colony-stimulating factor 1 (CSF1)], up to as high as 5,553-fold greater [in (MIP-1δ) / Chemokine (C-C motif) ligand 15 (CCL15)]; both of which are well-established pro-inflammatory chemotactic markers associated with immune system regulation by attracting additional inflammatory cells (i.e., neutrophils, monocytes, and lymphocytes) (69) (Table 2, last column). Three other markers (Angiogenin, Ck β 8-1/CCL23, and MIP-3α/CCL20) were also greater than 1,000-fold higher in the HCHF-fed mice relative to the HP-fed mice during the 15-week diet exposure but either downregulated or not significantly changed in the HP-fed mice from the baseline analysis (Table 1, 3rd column). To identify inflammatory mechanisms relevant to disease-associated biomarker networks, we performed pathway mapping analysis of the inflammation data for HCHF and HP diets, normalized to HP-diet levels at baseline (Table 2, 2nd and 3rd columns) using GeneGo software. Table 3 shows the five significant (*p* < 0.05) disease-relevant biomarker networks that could be influenced by this highly inflammatory HCHF dietary pattern, including pathways for highly inflammatory diseases such as arthritis, breast cancer, systemic lupus erythematosus, multiple sclerosis, and hepatitis.

**Table 3:** Diet and inflammation-associated disease-relevant pathways.

Biological Networks	*p*-value
Arthritis (core network)	0.0014
Breast neoplasm_Metalloproteases	0.0050
Lupus Erythematosus, Systemic (core network)	0.0087
Multiple Sclerosis (core network)	0.0104
Hepatitis (core network)	0.0383
Breast neoplasm_Inflammatory response	0.0891
Osteoarthritis (core network)	0.0930
Breast neoplasm_IL-6 and SP1	0.0930
Breast neoplasm_IL-2 and apoptosis	0.0988
Breast neoplasm_Chemokines	0.1065
Breast neoplasm_Anti-apoptosis	0.1387
Diabetes Mellitus, Type 1 (core network)	0.1609
Lung Neoplasms_Signal transduction	0.1645
Ovarian Neoplasms (core network)	0.1897
Fibrosis signaling: common features	0.2380
TLR signaling in airway epithelium	0.2530
Pulmonary Fibrosis	0.2665

Biomarker network pathway analysis was performed using GeneGo to associate with diet-dependent inflammation marker differences (see Table 2) between mice fed the high carbohydrate + high fat (HCHF) versus the high protein with higher fiber (HP) diet. IL, Interleukin and Toll-like receptor (TLR).

### Diet-dependent metabolic perturbation between mice fed HCHF or HP diet

**Figure 4:**
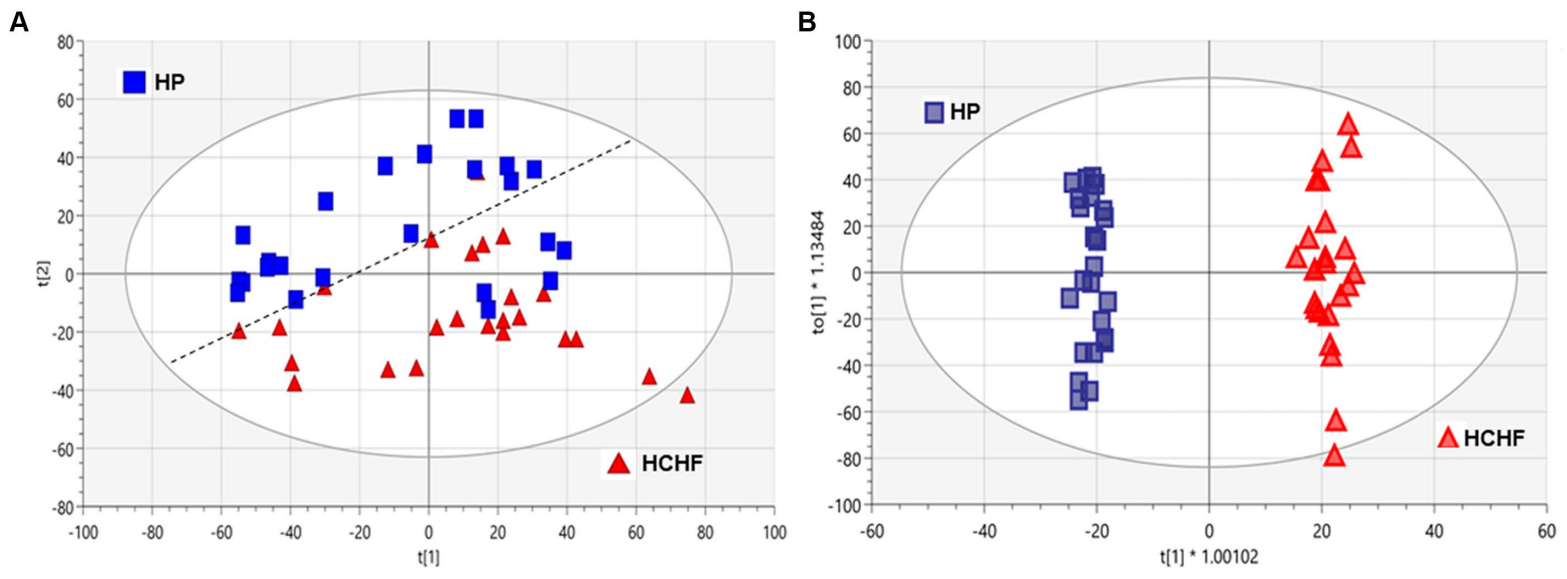
Multivariate analyses of HCHF versus HP diet-dependent metabolic responses in liver tissues. Principal component analysis (PCA) and orthogonal partial-least squares-determinant analysis (OPLS-DA) of metabolic responses after 15 weeks of differential diet exposures. Model statistics for **(A)** the PCA plot were R2X (1) = 0.226 and R2X (2) = 0.117, and for **(B)** the OPLS-DA plot were R2X(*cum*) = 0.432, R2Y(*cum*) = 0.989 and Q2(*cum*) = 0.916. HC, high carbohydrate; HF, high fat; HCHF, high carbohydrate + high fat; HCHF→HP, high carbohydrate + high fat crossed over to high protein with higher fiber; HP, high protein with higher fiber.

To better understand the influence of different dietary patterns on metabolic perturbation, we also performed untargeted metabolomics on the liver tissues. Unsupervised multivariate analysis was used to visualize distinct metabolic clusters that separated based on the dietary patterns (Figure 4A), and supervised multivariate analyses were used to determine metabolites contributing to significant differences, as shown in Figure 4B. The principle component analysis (PCA) and orthogonal partial-least squares-determinant analysis (OPLS-DA) demonstrated differentiation in metabolism for mice following 15 weeks of exposure between the HCHF versus the HP diets. Pairwise multivariate comparisons were conducted, and the variable influence on the projection (VIP) scores from the OPLS-DA (VIP ≥ 1.0), plus calculated *p-value (p* < 0.05) and fold change (FC ≥ 2.0), were used to determine signals that were most important to differentiate HCHF-fed mice and HP-fed mice. Signals that differentiated mice on the HCHF diet from the HP diet (1,133 signals) were prioritized for identification or annotation using our in-house physical standards library and public databases, and the results are reported according to an evidence-based ontology system (64) in Table 4. The table lists the differential diet exposures, which resulted in the annotation/identification of 74 unique metabolites that were perturbed between the HCHF-fed versus HP-fed mice. A positive fold-change indicates the metabolite is higher in the HCHF diet compared to the HP diet. Nucleosides (adenosine and ADP-ribose), bile acids (allocholic acid, glycocholic acid, glycylcholic acid, coprocholic acid, dehydrolithocholic acid, and taurocholic acid), acylcarnitines (acetylcarnitine, butenylcarnitine, and tiglylcarnitine), prostaglandins (16-Phenoxy tetranor prostaglandin F2.alpha. and 6-ketoprostaglandin F1.alpha.) and metabolites associated with folate metabolism (pterine and xanthopterin), and microbiome-tryptophan metabolism (5-hydrosindoleacetate, indole-3-ethanol, 3-indolepropionate, kynurenine, and indole-3-acetamide) were significantly lower in the HCHF mice compared to the HP mice; while lipids (sphinganine, D-Glucosyl-.beta.1–1’-D-erythro-sphingosin, Lyso-PAF C-18, butyl 9,12-octadecadienoate), cholesterols (27-hydroxycholesterol, 7-α,24(S)-Dihydroxy-4-cholesten-3-one), hormones (desogestrel), and metabolites associated with choline metabolism (S-adenosylmethionine and trimethylamine oxide) were significantly increased in HCHF mice compared to HP mice (Table 4).

**Table 4:** Identified/annotated metabolites perturbed between mice fed HCHF versus HP diet.

^1^Metabolite names	^2^Ontology	^3^Fold changes of *HCHF* vs. *HP*
Adenosine	OL2b	−2.3
ADP-ribose	PDa	−2.7
Pro Gly Asn	PDa	−38
5-hydroxyindoleacetate	OL1	−2
Allocholic acid	OL1	−3.9
Argininosuccinic acid	OL1	−2.1
Biliverdin	OL1	2.7
Glycocholate	OL1	−3.9
Indole-3-ethanol	OL1	−2.6
Pterine	OL1	−3.5
Sphinganine	OL1	2.3
27-Hydroxycholesterol	OL2a	3
3-Indolepropionate	OL2a	−2.3
3-Methoxytyrosine	OL2a	−6.9
3-Methyladenine	OL2a	−2.6
Butenylcarnitine	OL2a	−2.2
Coprocholic acid	OL2a	−4.1
Dehydrolithocholic acid	OL2a	−3.5
Homocysteine thiolactone	OL2a	2.4
Kynurenine	OL2a	−2.9
L-Tyrosine	OL2a	−5
L-Tyrosine isomer or derivatives	OL2a	−4
Nicotinamide	OL2a	−2.3
Normetanephrine	OL2a	−4.4
O-Acetylcarnitine	OL2a	−10.5
Prolyl-glycine	OL2a	2.8
S-adenosylmethionine	OL2a	81.4
Tiglylcarnitine	OL2a	−2.4
Tiglylcarnitine isomers or derivatives	OL2a	−2.9
Trimethylamine oxide	OL2a	7.4
Xylose	OL2a	−2.2
Xylose isomer or derviatives	OL2a	−2
3-methyladenine	OL2b	−15.3
Glycylcholic acid	OL2b	−4.1
Indole-3-acetamide	OL2b	−6.6
Propanoylcarnitine	OL2b	10.9
(+)-.alpha.-Tocopherol	PDa	7.6
(R) − 4-((3S,5R,8R,9S,10S,13R,14S,17R)-3-hydroxy-4,4,10,13,14-pentamethyl-7,11-dioxohexadecahydro-1H-cyclopenta[a]phenanthren-17-yl)pentanoic acid	PDa	-4
.alpha.-L-Glu-L-Tyr	PDa	2
1-(9Z-Octadecenoyl)-sn-glycero-3-phosphocholine	PDa	2.3
16-Phenoxy tetranor prostaglandin F2.alpha.	PDa	−5
4-Hydroxynonenal glutathione	PDa	−2.3
6-Ketoprostaglandin F1.alpha.	PDa	−2
6-Maleimidocaproic acid	PDa	−2.2
7.alpha.,24(S)-Dihydroxy-4-cholesten-3-one	PDa	3.6
Biliverdin	PDa	3.3
Biopterin	PDa	−2.5
Butyl 9,12-octadecadienoate	PDa	6.2
CAY10444	PDa	12.3
Cholest-4-en-26-oic acid, 7.alpha.-hydroxy-3-oxo	PDa	−25.7
D-erythro-C18-Sphingosine	PDa	2.3
Desogestrel	PDa	9.4
D-Glucosyl-.beta.1–1’-D-erythro-sphingosine	PDa	5.5
Glu Cys Leu	PDa	2.4
Glu Thr Phe	PDa	−5.6
Glu Tyr Asp Lys	PDa	−64.4
Glu-Ala-Lys	PDa	−3.6
Gly Ile Thr	PDa	−2.5
α- ± −amyrin	PDa	5.3
Ile Asn Gly	PDa	−3.6
Ile Val Ile	PDa	−18.3
Ile-Leu	PDa	−2.1
Lovastatin acid (Mevinolinic acid)	PDa	−8.4
Lys Leu	PDa	−2
Lyso-PAF C-18	PDa	2.6
LysoPE(15:0/0:0)	PDa	−2.4
N-Acetyl-L-arginine	PDa	−11.3
N-Oleoyl-D-erythro-sphingosylphosphorylcholine	PDa	2.1
Pilocarpine	PDa	–*
Protoporphyrin IX	PDa	2.1
Taurocholic acid	PDa	−85.7
Val Val	PDa	−2.1
Xanthopterin	PDa	−4.4
Ursocholic acid	OL1	
Glu Trp	PDa	

1 Metabolites satisfying VIP ≥1.0 and *p* < 0.05 and |FC| ≥ 2.0 in pairwise comparison of the HCHF vs. HP diet fed mice. A total of 1,133 peaks were differentiated between the pairwise comparison, with 74 identified or annotated with confidence (OL1, OL2a, OL2b, and PDA) via matching with an in-house physical standards library (IPSL) and/or public database (NIST or METLIN database).

2 Ontology levels: OL1, highly confident identification based on matching with via retention time (RT, with RT error ≤ |0.5|), exact mass (MS, with mass error < 5 ppm), and tandem mass similarity (MS/MS, with similarity ≥30); OL2a, confident identification based on matching with IPSL via MS and RT; OL2b, annotation for the isomer or derivatives of the compound listed but not the compound itself, based on matching with IPSL via MS and MS/MS; PDa, annotation based on matching with public database via MS and experimental MS/MS (could be the listed compound, or the isomer or derivatives of the listed compound).

3 Fold change, the ratio of intensity between the high carbohydrate + high fat (HCHF-) vs. high protein with higher fiber (HP) diet fed mice, based on the mean, indicates the direction and magnitude of perturbation: positive fold change (FC) indicates an increase in HCHF diet compared to HP diet and negative FC indicates a decrease in HCHF diet compared to HP diet. The lack of FC value (gray area) indicates that the metabolite did not satisfy the above criteria. –* indicates the metabolites were missing in the HCHF-fed mice.

During differential HCHF and HP dietary feeding, the corresponding diet-associated endogenous metabolic pathways that were perturbed in the mice were mainly associated with fatty acid and lipid biosynthesis/metabolism (6 pathways), thiamine metabolism (1 pathway), amino acid and glutathione metabolism (3 pathways), and taurine/hypotaurine metabolism (Table 5). The perturbation of thiamine metabolism was significant and more enriched than the others, followed by changes in lipid metabolism, specifically fatty acid biosynthesis (non-significant *p* > 0.05).

**Table 5:** Dietary pattern-differentiated metabolic pathways.

Metabolic pathway perturbed between HCHF *vs* HP	
*The Top 10 enriched metabolic pathways*	*p*-value
Thiamine metabolism	0.0410
Fatty acid biosynthesis	0.0879
Biosynthesis of unsaturated fatty acids	0.1042
Glycosphingolipid biosynthesis – ganglio series	0.1448
Lysine degradation	0.1782
Phenylalanine, tyrosine and tryptophan biosynthesis	0.1935
Taurine and hypotaurine metabolism	0.1957
Fatty acid degradation	0.2276
Glutathione metabolism	0.2349
Fatty acid elongation	0.2408

Metabolic pathways were enriched using MetaboAnalyst 5.0 with the mummichog and gene-set enrichment algorithms (GSEA). The listed *p*-values were calculated by integrating the mummichog and GSEA *p*-values using Fisher’s method (65, 66). The lower the *p*-value indicates greater pathway enrichment, even if it is non-significant.

## Discussion

Several studies have demonstrated the negative impacts of *Westernized* or HCHF dietary patterns on overall health, typically patterns high in simple carbohydrates (including refined sugars, grains, and syrups), saturated and n-6 polyunsaturated fats (17, 31, 70), *trans*-fats, and ultra-processed foods (14, 37). The HCHF diets can particularly drive chronic inflammation and metabolic disruption associated with immunosuppression and disease progression (18, 71–73). Chronic inflammation alone affects many individuals by contributing to the etiology and progression of most chronic diseases, including arthritis, cancer, and musculo-skeletal and liver-based conditions, as also revealed in our study (Table 3), and has been attributed to more than 50% of all deaths globally (27, 28). By comparison, Hebert and colleagues have provided an informative perspective by reviewing many dietary patterns and associated dietary indexes that relate the influence of specific foods and nutrients to chronic inflammation and resultant immune dysregulation alone or in combination with metabolic dysfunction (26). However, this level of nutritional information has largely not been strategically integrated into individualized medical practice, where emerging precision nutrition approaches could co-assist established methods of treatment and help clinicians deliver personalized medicine for more holistic disease prevention, reduction in co-morbid disease risks, and better management of disease complications that are strongly associated with sub-optimal dietary practices. Various animal and human studies have demonstrated the benefits, for the most part, of dietary patterns with higher protein content (at ranges between 23 and 69% of total calories) on slowing or inhibiting chronic inflammation and normalizing metabolic perturbations toward homeostasis (16, 48, 74, 75). However, the application of the research is complicated because the most recent guidance for daily protein intake, being between 10 and 35%, is nearly 15 years old (76) and still too broad for the general patient to know *how much is personally best* within that range, or if it is better for them to consume more. Similarly, the advice for total carbohydrate and fat consumption also varies widely, at 45–65% and 20–35%, respectively (54, 61), but more work has been done to better specify which types of carbohydrates (complex instead of simple/refined) and fats (poly-unsaturated instead of trans- and saturated) are most beneficial. The HP diet was specifically designed to have higher fiber (complex carbohydrate) content, as a primary study goal was to compare a healthier dietary pattern (HP with higher fiber) to an unhealthy pattern (HCHF). Our overall research seeks to identify dietary patterns with optimized macronutrient content that result in healthy body weight management, glucose homeostasis, low-to-no inflammation, and decreased risk for the development of chronic diseases such as cancer, type II diabetes, or cardiometabolic outcomes for women after menopause when the prevalence of such conditions increases (77–79). We believe the HP pattern studied here demonstrates such benefits in a synergistic manner. However, the goal of these initial pilot studies was to demonstrate an effect of better responses by comparing them primarily with the HCHF dietary pattern. In future studies, we plan to evaluate an HP formulation with identical inulin kcal% content and high protein alone to the HC, HF, and current HP formulation, all with the higher inulin content, to determine the impacts of the increased fiber content. We also plan to determine whether changing the protein source (animal- vs. plant-based) for the HP dietary pattern will further optimize it and lead to better outcomes in this model.

This current study used an ovariectomized mouse model to mimic the postmenopausal state in women to determine the impact of differential macronutrient compositions (carbohydrate vs. fat versus protein) in different dietary patterns (high carbohydrate-HC, high fat-HF, high carbohydrate-plus-high fat-HCHF, and high protein with higher fiber/complex carbohydrate-HP) on body weight changes and glucose levels, for 15 weeks. In the 4-diet study, we switched a group of mice being fed the HCHF diet to the HP diet after 12 weeks to mimic a 21-day diet intervention/modification to determine if a significant change in body weight would occur. In addition to the significant changes in body weight between the HF, HCHF, and HCHF→HP groups of mice versus the HP-fed group, there was also a significant difference in the weight of HF-fed mice compared to the HC-fed mice (~39 g vs. ~32 g). Here, we are comparing the impact of 45/25% of fat/simple carbohydrate in the HF pattern to 25/45% of fat/simple carbohydrate in the HC pattern, and both with 30% protein. The results closely recapitulate what many diet-associated studies for obesity changes have shown, being that at these or similar proportions, a diet higher in fat content will result in obesity while a diet with reversed proportion of carbohydrates will not be to a point (80–84). While the results confirm the former study, it is important to note here that the blood glucose level findings may seem the opposite. Again, the HF diet rendered higher glucose in mice after 15 weeks, but somewhat unexpectedly, mice fed the HC diet ended up with the second highest levels of blood glucose after 15 weeks, without the corresponding significant increase in body weight as compared to the mice fed the HCHF dietary pattern. As mentioned in the Introduction section, we believe this result fundamentally recapitulates dietary influence on glucose metabolism and the etiology of insulin resistance and metabolic syndrome in the absence of obesity (or before development), which reflects the onset or incidence of type II diabetes in non-obese individuals (51, 52). It is well-documented that overconsumption of dietary fat can result in elevated and accumulating levels of lipids that then interrupt normal cell signaling and trigger pro-inflammatory immune cell infiltration, leading to chronic inflammation. Once this vicious cycle begins, it further exacerbates glucose metabolism from homeostasis regulated by the liver and pancreas (53). Regarding the overconsumption of simple carbohydrates that metabolize rapidly and break down to sugar (glucose), the results of the HC diet are suggestive of decreased insulin sensitivity, where the amount of carbohydrates is too much for the body to maintain homeostasis. We have currently measured glucose levels at four timepoints for the four-diet pilot study (Figure 3), but plan to evaluate changes in both insulin and leptin levels in stored plasma biospecimens to provide a better understanding of how these different dietary patterns are impacting biological processes linked to the chronic condition of type II diabetes outcomes in this postmenopausal model.

We followed up with a partial reproducibility study in the same mouse model but without a diet switch, only comparing the most divergent HCHF and HP dietary patterns based on body weight results to further interrogate diet-associated impacts on the mechanisms of inflammation and metabolism. Again, we used liver tissues to represent systemic changes, as it is the primary metabolic organ. The HCHF diet resulted in the most dramatic gains in body weight, compared to the other three dietary patterns, though not significantly different by the end of the initial study from mice fed the HF diet. Of note, although mice were randomly assigned to each diet group, we did find that following quarantine, the five heaviest mice were all placed in the HCHF→HP diet intervention group. On four separate days, this likely explains the fact that this group of mice consumed a significantly larger amount of food (Figure 2A), even compared to their counterpart group of mice that were maintained on the HCHF diet for the duration of the study. It may be presumed that we would have seen even greater weight loss following the diet conversion had the distribution of the mice by initial weight been more uniformly *random*, as it was at the beginning of the two-diet study. In a reproducible manner, mice on the HCHF diet also consumed more food in general in the two-diet study, but these results are consistent with literature studying high-fat diet macronutrient content (58, 85, 86). The average total body weight of mice on the HCHF diet was ~1.4x that of mice fed the HP diet at week 15 (Figures 2B,D), without an overall significant difference in food consumption (Figures 2A,C), other than for the HCHF→HP mice following the first week of the intervention. Although our HCHF diet has 40% matched total carbohydrate (simple) and fat composition, which is lower than many “high carbohydrate only” or “high fat only” research-based diets (~45–70%), it interestingly and synergistically recapitulated Western-style diet-associated phenotypes, which we believe are directly related to its high inflammation-inducing properties (87, 88).

A vast body of work has established critical associations between diet and inflammation, which specifically influence a myriad of alterations in cytokine/chemokine/adipokine networks (10, 16, 18, 42, 44, 71). However, the field is gaining more knowledge every day, especially related to diet associations and obesity, driven by and reciprocally causing disruptions in mechanisms such as inflammation and metabolism. Here, we showed that our HCHF diet induced significant upregulation in 80 inflammation markers compared to our HP diet after 15 weeks in the postmenopausal mouse model. Our disease-relevant biomarker pathway analysis results demonstrated that the dietary pattern we modeled had significant implications for several chronic diseases (Table 3). Although some of the elevations are likely age-associated, massive inflammatory alterations clearly occur following HCHF diet exposure (Table 2), presumably resulting in significant immune system dysfunction. While this alone is a dramatic dietary impact, having all markers become upregulated does present a challenge for data interpretation, as most of the markers analyzed have pleiotropic activity, and a disease context will likely be the most informative foundation to whether the marker is acting in a pro-inflammatory or anti-inflammatory manner. For example, consider what our data show regarding the potential impact of the HCHF diet on fostering breast cancer, as breast neoplasm via inflammatory metalloprotease(s) mechanisms was the second most significantly indicated disease-relevant pathway, based on a biomarker (cytokines) network analysis (Table 3). In addition, several other breast neoplasm_inflammatory mechanisms were on the list, though not indicated at the threshold of significance. The cytokine array includes data for two such proteins, TIMP-1 and TIMP-2 (Tissue Inhibitor of Matrix Metalloproteinases-1/2), which are endogenous inhibitors of the extracellular matrix-degrading proteins called matrix metalloproteinases (MMPs). In normalcy, the balance of TIMPs and MMPs plays a role in removing injured cells/tissue, but in cancer, proteins such as MMP-2 are critical for cancer cell metastasis (89). Thus, the balance between their expressions is essential to tissue integrity and immune homeostasis. Increased levels of TIMP-1 (1617-fold) and TIMP-2 (188-fold) in response to the HCHF diet exposure suggests that there was a corresponding initial systemic increase in MMP-1 and -2 (though not directly measured by our array) to restore balance and prevent tissue damage. This type of tissue damage could also occur in some of the other implicated chronic diseases, including arthritis, multiple sclerosis, and hepatitis. Compared to the HP diet, where TIMP-1 was only increased 51-fold and TIMP-2 was not significantly changed, our data suggest that MMPs (1 and 2) were not elevated as much or at all in mice that consumed the HP diet, and the HP dietary pattern is far less inflammatory compared to the HCHF dietary pattern. Interestingly, Fjaere and colleagues previously demonstrated that TIMP-1 is required for inducing glucose intolerance and hepatic steatosis on high-fat and/or high-fat plus high sucrose content diets (90). In addition, MIP-1δ, also known as chemokine (C-C motif) ligand 15 (CCL15), was the most highly elevated cytokine in livers of mice fed the HCHF diet relative to the HP diet and plays an essential role in regulating endothelial cell differentiation, angiogenesis, and biosynthesis of other cytokines related to these functions; and thus could also explain why Breast neoplasm diseases and hepatic-related diseases were highly indicated as impacted by the HCHF dietary pattern (91–94).

Overall, HCHF-fed mice had higher cytokine levels compared to HP mice after 15 weeks of diet exposure, suggesting a dramatic shift toward a chronically inflamed state by comparison. Gross impacts on body weight gain leading to a diet-dependent obesogenic environment further complicates the matter by inducing some of the context-dependent dualistic activities many of these molecules possess (e.g., IL-6, IFN-γ, MCSF, and VEGF) (30, 95–97). Even so, several markers in the array panel do contribute primarily to pro-inflammatory mechanisms (i.e., IL-1α/β, IL-6, IL-12, MCP-1/2/3/4, MCSF, RANTES, Leptin, Angiogenin, HGF, IGFBPs, Osteopontin, and TIMP-1), while others facilitate anti-inflammatory or resolution mechanisms (i.e., IL-10, IL-13, IP-10, LIF, PIGF, and TGF-β2) under conditions of a “normal” immune responses. Again, the contrast in response was clear with consumption of the HP diet, where significantly fewer pro-inflammatory markers were upregulated, several were instead downregulated, and key anti-inflammatory markers (i.e., IL-10, IL-13, LIF, and TGF-β2) were upregulated. Further investigation is needed to determine a greater range of dietary components that directly link the mechanism of inflammation to obesity and then to resultant chronic disease conditions, where impairment of the immune system diminishes the body’s ability to respond to any insult or injury, and sets the stage for disease progression, based on known roles for chronic inflammation in their etiologies (45).

Regarding impacts on metabolism demonstrated by the two-diet study, the HCHF diet compared to the HP diet significantly perturbed endogenous pathways, reflecting a breakdown in multiple nutritionally relevant biosynthetic pathways (Figure 4; Tables 4, 5). As expected, there were disruptions in lipid metabolism, specifically for fatty acid synthesis, degradation, and elongation, as well as glycosphingolipid biosynthesis in mice fed the HCHF diet. Thiamine metabolism was the most significantly perturbed metabolic pathway, even though thiamine itself was not identified as a significantly differential metabolite during annotation. Thiamine, or Vitamin B1, is a water-soluble essential micronutrient because the liver can only store a small amount. It plays critical roles in food metabolism for energy (glucose metabolism), maintains the growth and functionality of several cell types, and is required for a healthy nervous system (98). It is likely indicated in our study due to the established relationship between thiamine deficiency and obesity, and deficiency can cause several dysfunctional conditions in the liver (13, 99–101). The HCHF diet also decreased taurine and hypotaurine metabolism relative to the HP diet, though not significantly in our pathway analysis; we make mention of it because it is an observation previously shown in a diabetic study feeding a high fat-high sucrose diet to male C57 mice (50, 102). Taken together, these observations are encouraging because our model has identified targetable, nutritionally relevant mechanisms that can likely be reversed with dietary pattern modification.

## Conclusion

Our study used high-throughput and *omics* approaches to identify diet-driven, inflammation, and metabolic markers and pathway perturbations in response to differential dietary macronutrient contents in a postmenopausal mouse model. Overall, our data suggest little to no adverse impacts on body weight from the HP diet exposure, whereas only HF and HCHF diet-exposed mice started gaining significantly more weight after 2 weeks and maintained the condition of obesity by the end of 15 weeks. This body weight gain was shown to be modifiable following a diet cross-over intervention from the HCHF diet to the HP diet for 21 days. We anticipate that if the study continued for a longer time, allowing mice to fully adjust to the new diet and begin consuming similar amounts of this healthier food, it would show the obesity phenotype could be completely reversed with this dietary intervention. Regarding inflammatory response changes, the HP dietary pattern increased the level of 24/80 cytokines and decreased 8/80 cytokines significantly after 15 weeks. In contrast, the HCHF dietary pattern significantly upregulated 79/80 cytokines over the course of the study, compared to baseline and all 80 markers in a comparative analysis with the HP diet. Furthermore, differential changes in metabolism suggest that healthier dietary patterns cause less perturbation in key regulatory endogenous pathways. Our study has increased the understanding of macronutrient responses in two key mechanisms linked to a multiplicity of detrimental health outcomes, and it is particularly relevant to the influence of different dietary patterns on the health outcomes of postmenopausal women. This model provides an important tool to identify biomarkers for monitoring health status considering total dietary macronutrient composition, and this research tool will allow us to test modification strategies for optimizing nutrition linked to relevant disease-associated phenotypes that could inform trials designed to improve outcomes for women’s health.

## Data availability statement

The datasets presented in this study can be found in online repositories. The names of the repository/repositories and accession number(s) can be found at: https://www.metabolomicsworkbench.org, Project ID (PR001044) and Study ID (ST001635).

## Ethics statement

The animal study was approved by the Institutional Animal Care and Use Committee at Explora BioLabs (San Diego, CA), a subsidiary of Charles River Laboratories and the University of North Carolina at Chapel Hill Institutional Animal Care and Use Committee. The study was conducted in accordance with the local legislation and institutional requirements.

## Author contributions

Y-yL: Data curation, Formal analysis, Methodology, Visualization, Writing – original draft, Writing – review & editing. SSM: Data curation, Project administration, Validation, Writing – review & editing. ER: Data curation, Project administration, Supervision, Writing – review & editing. CS: Data curation, Project administration, Supervision, Writing – review & editing. HF: Data curation, Formal analysis, Writing – review & editing. JP: Formal analysis, Validation, Visualization, Writing – original draft, Writing – review & editing. SLM: Formal analysis, Software, Validation, Writing – review & editing. WP: Data curation, Formal analysis, Validation, Visualization, Writing – original draft, Writing – review & editing. SH: Funding acquisition, Investigation, Methodology, Resources, Supervision, Writing – review & editing. SS: Funding acquisition, Investigation, Methodology, Resources, Supervision, Validation, Writing – review & editing. DS: Conceptualization, Data curation, Formal analysis, Funding acquisition, Investigation, Methodology, Project administration, Resources, Supervision, Visualization, Writing – original draft, Writing – review & editing.

## Author’s Note

Queries regarding metabolomics data acquisition should be directed to SS. All other queries should be sent to DS.
